# Quantifying the evolution of flow boiling bubbles by statistical testing and image analysis: toward a general model

**DOI:** 10.1038/srep31548

**Published:** 2016-08-16

**Authors:** Qingtai Xiao, Jianxin Xu, Hua Wang

**Affiliations:** 1 Kunming University 5 of Science and Technology, State Key Laboratory of Complex Nonferrous Metal Resources Clean Utilization, Kunming, 650093, China; 2 Kunming University of Science and Technology, Faculty of Metallurgy and Energy Engineering, Kunming, 650093, China; 3 Kunming University of Science and Technology, Quality Development Institute, Kunming, 650093, China

## Abstract

A new index, the estimate of the error variance, which can be used to quantify the evolution of the flow patterns when multiphase components or tracers are difficultly distinguishable, was proposed. The homogeneity degree of the luminance space distribution behind the viewing windows in the direct contact boiling heat transfer process was explored. With image analysis and a linear statistical model, the F-test of the statistical analysis was used to test whether the light was uniform, and a non-linear method was used to determine the direction and position of a fixed source light. The experimental results showed that the inflection point of the new index was approximately equal to the mixing time. The new index has been popularized and applied to a multiphase macro mixing process by top blowing in a stirred tank. Moreover, a general quantifying model was introduced for demonstrating the relationship between the flow patterns of the bubble swarms and heat transfer. The results can be applied to investigate other mixing processes that are very difficult to recognize the target.

The performance of a mixing process can be characterized by the blending time, which is a very important parameter and is also called the mixing time. The shorter the mixing time, the more effective the mixing^1^. Accurate characterization of uniform mixing is not only essential for the optimization of the heat transfer process and reactor design but can also precise control of the reaction time.

A large number of experimental works have been devoted to studying this issue. The mixing time methods consist of the box-counting with erosion method^2^, the thermal method, the conductometric method, the pH method, the decolorization methods, the Schlieren method, etc. However, there is no universally accepted method, mainly because each local and global measurement method has its own limitations, the details of which have been reported by Cabaret *et al*.^3^. The Betti numbers method was recently proposed to characterize the gas-liquid-solid three-phase mixing effects based on the reaction of CH_4_ and ZnO with a molten salt system^4^. In terms of the bubbling regimes in direct contact heat exchanger (DCHE), Betti numbers can be used to estimate the number of bubbles aggregating in flow patterns and to obtain the pseudo-homogeneous time^5^. We concluded that bubble swarm images can be captured and processed advantageously due to image enhancement technology and mathematical methods^6^.

Digital pictures may be taken at different exposures and lighting, so the homogeneity degree of the luminance space distribution must be taken into consideration in terms of the image analysis or visual perception. In general, after the illuminated area accepts the light, the luminance level differs in various observation directions because of the different reflection characteristics of the material. This problem is one of the most challenging topics in image analysis applications.

Some studies in the literature note the effects of the luminance distribution. On the one hand, the luminance distribution also appeared in the literature regarding mixing fields. The image analysis method can be used to obtain the mixing time in a discontinuous powder mixer, which has been studied by Daumann and co-workers^7^. The work from Yeoh *et al*.^8^ clearly demonstrated that the large eddy simulation technique coupled with the sliding-mesh methodology can be used to characterize the transient mixing state in a stirred vessel and to provide a very detailed evolution pattern of the concentration field in space and time during the process. On the other hand, the luminance distribution also appeared in the literature regarding building fields. For the purpose of finding ways to control glares coming from windows, as in the case of glare sources in the daylight that have a non-uniform luminous distribution Kim *et al*.^9^ studied the difference in glare sensations between uniform and non-uniform glare sources, and Kim and co-workers^10^ developed an approach to identify the parts of the window where the glare is generated when the window has a non-uniform luminance distribution. Furthermore, the luminance distribution, as a determinate, appeared in the literature regarding food fields. Wada *et al*.^11^ and Arce-Lopera *et al*.^12^ found that the appetizingly fresh appearance of vegetables or our visual perception of their freshness is highly influenced by the luminance distribution according to the evidence from an image analysis of a cabbage leaf and other variables, such as spatial patterns. The mixing time and light intensity for the comparison of the photobioreactor design considering biohydrogen production is the key issue according to the study by Oncel *et al*.^13^.

Recently, linear and nonlinear regression models have been used to investigate a 662 × 621 gray image of a rug^14^. Notably, Markou *et al*.^15^ applied the multivariate statistical methods of factor analysis and cluster analysis on sky luminance scan data, whereas skylight was studied by Dumortier *et al*.^16^. Additionally, as shown by Coent *et al*.^2^, Xu *et al*.^4^, Kim *et al*.^9^,^10^, Demidenko^14^, Huang *et al*.^5^ and Fei *et al*.^6^, image analysis with advanced statistical methods (concerning mathematical knowledge), regarded as a normal practice, is gaining importance for object identification. However, studies on illuminance and luminance using image technology and a statistical approach do not appeared widely in the international literatures to date. In addition, the studies of heat-mass transfer law and how to enhance it are challenging topics in direct contact heat transfer. Inspired and motivated by Huang *et al*.^5^, Demidenko^14^ and Markou *et al*.^15^, statistical models for the gray images of the bubble swarm in a DCHE based on the gray scale distribution (or simply, gray distribution) are developed. From the viewpoint of experimental analysis, the heat transfer processes can be elucidated.

## Experiments and Methodology

### Experimental apparatus and design of experiments

**Figure 1:**
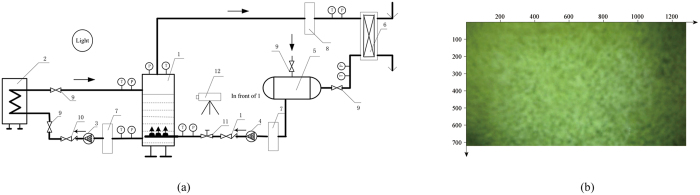
Real experimental data acquisition: (**a**) is the experimental equipment for testing of platform and (**b**) is a sample image chosen from patterns in the mixing process.

The schematic of the experiment conducted in the present study is shown in Fig. 1a. There are twelve main experimental pieces of equipment, including a direct contact evaporator (1), electric heater (2), gear oil pump (3), centrifugal pump (4), storage vessel (5), plate condenser (6), liquid mass flow-meter (7), gas mass flow-meter (8), stop value (9), check valve (10), manual modulation value (11) and camera (12). There are two circulation loops in the test device for this experiment, the continuous-phase circulation loop for fluid flow and the dispersed-phase circulation loop for the working medium flow. The heat transfer fluid (HTF) and refrigerant R-245fa (1, 1, 1, 3, 3 pentafluorogropane) were used as the continuous phase and dispersed phase in all runs, respectively. As listed by Huang *et al*.^5^, design parameters with four factors were selected to investigate the influence of the heat transfer capacity. The height of the HTF in the DCHE was measured by a dipstick; the initial heat transfer temperature difference was obtained using a K-type thermocouple at the temperature measuring holes; and the flow rate of the refrigerant and HTF were regulated via the frequency control cabinet. The designing of the experimental plan affecting the heat transfer capacity of the tested DCHE was determined through the orthogonal array experimental design method, namely the L_9_(3^4^) orthogonal array table, which is suitable for an experimental design with four factors and three levels. According to the orthogonal array table, the numbers L_1_-L_9_ denote different experimental levels.

According to the design of the experiments and the green image (Fig. 1b), it seems clear from visual inspection that light is coming from the upper left. However, the accurate position of the light source was unknown, which has a significant impact on the implementation of uniform lighting.

### Methodology

Generally, images may be divided into roughly two groups, structured and unstructured. Structured images are complex, and unlike unstructured images, a multinomial distribution for gray levels may serve as a uniform probabilistic model^14^. In the present research, the bubble swarm image is structured or content-dependent because the bubbles move in a stochastic manner. Thus, a gray-distribution model, which assumes that the gray scale level values are the same type of object, can be used to differentiate bubble swarm from the captured flow image. Other researchers (Coent *et al*.^2^, Huang *et al*.^5^ and Fei *et al*.^6^) also investigated the flow pattern by image analysis. However, they all extracted and employed the binary information. This work brings new insights, namely, statistical image analysis, to the study flow pattern.

#### Coordinate transform

Before modeling the gray scale level matrix (or simply, gray matrix) *M*, there are three important steps.

**Step 1** transform the bubble patterns from the color image to *M* so that the frame coordinate system can be set up.

**Step 2** set the row number of *M* as the abscissa and set the rank number of *M* as the ordinate.

**Step 3** treat the increasing direction of the row and rank numbers of *M* as the positive direction of the coordinate axis.

As shown in Fig. 1b, a 720 × 1280 image representing one piece of the bubble patterns was randomly chosen, and it was contaminated with light in a coordinate system with the origin (1, 1) at the top-left corner. As seen in the above section, the light comes from the direction of the origin, according to the experiment design.

#### Multiple linear regression model

In the present statistics analysis, multiple linear regression is an approach for modeling the relationship between a scalar dependent variable *M*(*u*, *v*) and two explanatory variables (or independent variables), denoted *u* and *v*. On the basis of the new coordinate system and taking the vec operator, the following linear regression model is defined as:


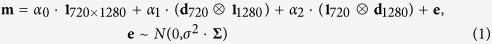


where **d**_720_ = (1, 2, ··· . 720)′, **d**_1280_ = (1, 2, ··· . 1280)′, **1** is the column vector of 1s of the respective dimension, ⊗ indicates the matrix Kronecker product, **m** = vec(*M*) is the *n* = 720 × 1280 × 1 = 921600 vector, and *σ*^2^ is unknown but the co-variance matrix **Σ** is known and non-singular. That is, the gray scale level (scalar dependent variable) is considered to be a linear function of the pixel coordinates (explanatory variables). The relation may be viewed as a planar regression where *α*_0_ is the intercept, the light intensity at the upper-left corner; *α*_1_ is the slope coefficient, the rate at which the light intensity increases or drops vertically; and *α*_2_ is the rate at which the light changes horizontally. It is clear that if the gray distribution is uniform, *α*_1_ = *α*_2_ = 0.

In model (1), *σ*^2^ ⋅ **Σ** is the co-variance matrix of the error term **e**. The error variance *σ*^2^ is a constant parameter but unknown actually. Fortunately, the unknown parameter can be estimated by sample data that transformed from every image, namely, the estimator of *σ*^2^ is *S*^2^. In a word, the new index *S*^2^ means a estimator of a measure of how much the two independent random variables, the abscissa *u* and the ordinate *v*, change together. Hence, this new index was extracted from the multiple linear regression model and can be proposed for quantifying the flow patterns or employed it for quantitative characterization of mixing effect by color or gray images directly in experiments. Simply, *S*^2^ will be greater if there are more intimate mixtures in the RGB or gray image and *S*^2^ will be smaller if tracer particles are few and scattered distribution.

#### *F*-test

It is easy to test the significance of these regression coefficients *α*_1_ and *α*_2_, called *F*-test hereafter for testing whether the gray distribution is uniform. Define two residual sums of squares:


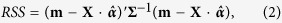



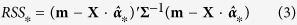


where *RSS* is the absolute minimum of the weighted least squares (WLS), 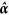 is the generalized least squares (GLS) estimate of ***α*** = (*α*_0_, *α*_1_, *α*_2_)′, **X** = (*x*_*ij*_) = (**1**_1720×1280_, **d**_720_ ⊗ **1**_1280_, **1**_720_ ⊗ **d**_1280_) is the 921600 × 3 design matrix of full rank, RSS_*_ is the residual sum of the WLS under the restriction *α*_1_ = *α*_2_ = 0 and 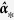 is the GLS estimate under the same restriction. Then, the *F*-test is as follows:


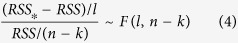


#### Estimating the light direction and position

As follows from the basic laws of optics and model (1), the nonlinear regression model for estimating the light direction and position is given by


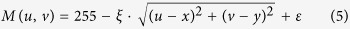


where *ξ* is the absorption coefficient, (*x*, *y*) is the position of the source of light, (*u*, *v*) is the element of **M**, and *ε* is the error term.

In summary, the hypothesis-testing approach has three advantages. First, the multiple linear regression model can be used to quantify the tendency of the flow patterns and obtain the mixing time for evaluating the mixing effect. In particular, whether the multiphase components or tracers are visually distinguishable or the mixture images are difficult to convert into a black-white image to obtain a binary image by applying an appropriated grey level threshold for the colour video images, the new index *S*^2^ can be used to measure mixture uniformity and mixing efficiency. In addition, *S*^2^, along with other methods, can also be summarized by a general expression in the section “General model” to investigate the relationship between the flow pattern and heat transfer. Second, *F*-test can be used to test whether the lighting is uniform according to the gray distribution. Third, the nonlinear regression model can be used to estimate where the light comes from, and it can be further used to estimate the accurate position of the light source.

## Experimental Results and Discussion

### Validation of the statistical approach

#### Result of *F*-test

To test whether the piece of patterns in Fig. 1b has a uniform gray distribution, we test the null hypothesis *H*_0_ : *α*_1_ = *α*_2_ = 0 using the image process technology for translating an image to a gray matrix and the *F*-test method for obtaining the objective criterion. Applying ordinary least squares (OLS), it is found that the regression-estimated equation is 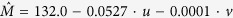, *F* statistic is *F* = 1.0313 × 10^5^ and that the *P*-value is *P* ≈ 0. Thus, the hypothesis that the gray distribution is uniformly distributed is overwhelmingly rejected. However, the test may be conservative.

#### Light direction and position

As follows from this regression 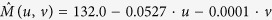, the minimum average gray scale level is 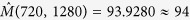, corresponding to the bottom-right corner of the bubble image, whereas the maximum 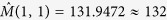 corresponds to the top-left corner of the bubble image. Moreover, it is easy to estimate where the light comes from according to the positive or negative sign of the coefficients *α*_0_ and *α*_1_. Because the first slope coefficient is negative and the second is negative, the light comes from the top-left corner. It was included that the estimating result of light source direction is consistent with visual inspection. Estimating parameters of this model (5) by nonlinear regression gives *ξ* = 0.1816, *x* = 1.2276, and *y* = 1.4920. This means that the estimated location of the light is (1.2276, 1.4920). That the valid coordinate values can indicate the accurate position of the light source is the main merit of the proposed method.

**Figure 2:**
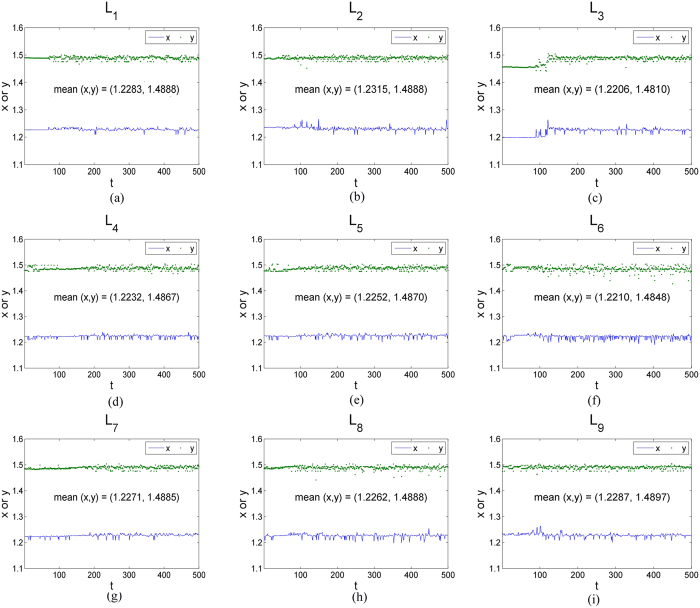
500 light source coordinates (*x*, *y*) with time obtained from images taken regularly from the beginning of mixture to the end in each experimental case L_1_–L_9_ and the values of coordinates smoothly oscillate about their averages (1.23, 1.49).

The nonlinear regression model was employed to estimate the light source position (*x*, *y*). The light direction and position of all cases are shown in Fig. 2. 500 light source coordinates (*x*, *y*) with time were obtained from images taken regularly from the beginning of mixture to the end in each experimental case L_1_–L_9_. The light source coordinates of nine experimental tests have the average of *x* = 1.23, *y* = 1.49 with the standard deviation of 0.36% and 0.27%, respectively. Hence, the accurate location of the light source was determined and expressed by coordinate values in a coordinates system. The direction of the light source was the top left since the horizontal and vertical ordinate are both positive. This was validated by the previous design of experiments and cannot be obtained by other three existed methods. Interestingly, the linear and nonlinear models give the same light direction.

#### A new index

Furthermore, the estimate of the error variance *σ*^2^ is *S*^2^ = 536.5847. In fact, the quantity of bubble swarms was very small at the beginning of the mixing process, whereas it was very large at the uniform mixing process. *S*^2^ is applied to represent the difference between what is explained by the systematic part of the model and what is observed. The reason why *S*^2^ experienced an ascending transition change is mainly because of the growing number of bubble swarms.

**Figure 3:**
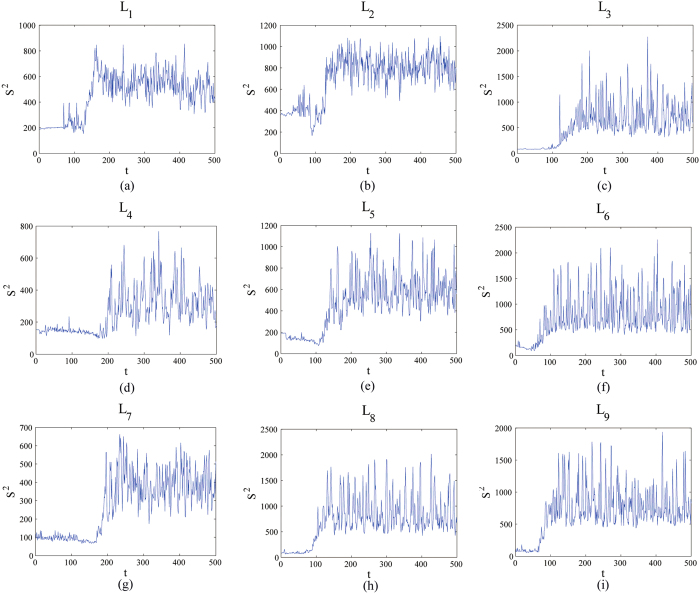
Evolution curves of the estimate of error variance, *S*^2^, in the mixing system for direct contact heat transfer and representative experimental design.

According to the orthogonal array table L_9_ (3^4^), there are nine experimental cases for testing the presented method, and every case consists of 500 flow images. The Betti numbers method^5^ and the UC method^6^ have also been tested with the same samples. The plots of *S*^2^ versus the experimental time *t* (time unit: seconds) obtained from the red component of the color video images taken regularly from the beginning of mixture to the end are shown in Fig. 3. An interesting tendency occurs in every case among L_1_–L_9_. A homogeneity is achieved after some seconds which can be regarded as the evaluation criterion to quantify the evolution of the flow boiling bubbles.

**Figure 4:**
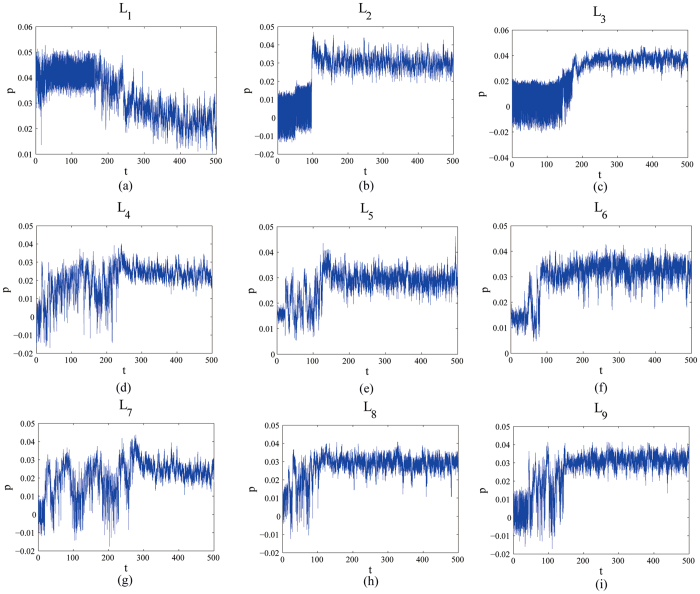
The evolution of this slope *p* with time obtained from images taken regularly from the beginning of mixture to the end by the box-counting with erosions method: *p* increase at the beginning and then rapidly becomes stabilized after fluctuations.

In Fig. 4, it is clearly observed that the slope *p* with time obtained from images taken regularly from the beginning of mixture to the end by the box-counting with erosions method increase at the beginning and then rapidly becomes stabilized after fluctuations in every case, which, as expect, are similar to those of *S*^2^ represented in Fig. 3. However, the box-counting with erosions method is not available for testing for uniform lighting and estimating light direction and position. In addition, the method is not useful to quantify the evolution in some special complicated experimental case according to the experimental case L_1_ (Fig. 4). Finally, the binary images were employed to calculate the slope *p* in Fig. 4 whereas the RGB images were employed to calculate *S*^2^ in Fig. 3.

All of the inflection points *t*_*s*_, namely the mixing time, are obtained using the same approach as Xu *et al*.^4^. Clearly, *t*_*s*_ is approximately equal to the mixing time *t*_*p*_, *t*_*B*_ and *t*_*UC*_, which were obtained from the boxing-count with erosion method (Fig. 4), the Betti numbers method^5^ and the UC method^6^, respectively (Table 1). Hence, the presented method has been tested with many cases and validated with other methods.

Moreover, the parameters *t*_*s*_ and 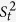, which can be used to characterize a bubbling flow pattern with non-uniform luminance, are related to 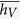. Next, a model is constructed to note the relationship.

#### Quantifying the synergy

**Figure 5:**
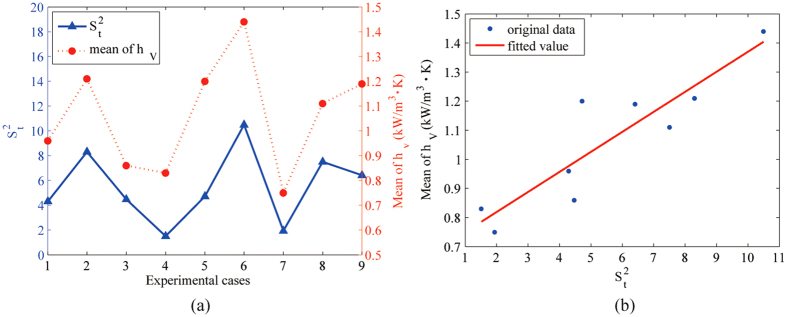
Synergic relationship between the average volumetric heat transfer coefficient 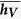 and the parameter 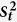, combined with *t*_*S*_ and 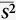: 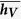 is relatively high when 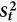 is high and 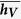 is relatively low when 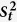 is low; the linear relationship is illustrated with a correlation coefficient of 0.9011.

Let 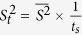, where 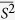 is the average of *S*^2^ of the uniform mixing process influenced by light. Clearly, in Fig. 5a, the tendency of 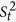 is consistent with that of 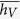. According to our analysis, a linear relation between 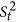 and 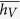 seems to be an outcome. Given 
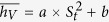
, the least squares fitting method was used to obtain the parameters *a* and *b*. In this work, *a* = 0.0689, *b* = 0.6812, the correlation coefficient is 0.9011 and the determination coefficient is 0.8120. The linear relationship is illustrated in Fig. 5b.

#### Effect on the spatial resolution

To address the effect of the camera spatial resolution on the statistical approach, the same experiment bubble piece of patterns was used to record where image processing is performed at decreasing resolution. It was observed that the *S*^2^ evolution is remarkably superimposed, indicating that the *S*^2^ evolution does not entirely depend on the image size. The *F*-test parameters for the different image resolutions considering the same experiment bubble piece of patterns are presented (Table 2). In the last column of the table, it is found that the estimated direction of the light is all upper left (Table 2). It also can be seen that the image resolution does not have a significant influence on the results if adequate equipment is used (Table 2).

### General model

In the previous section, effort was made to quantify the synergy of luminance space distribution and heat transfer performance in a DCHE. The former is one manifestation of bubble swarm patterns. The objective of this section is to build a general model for quantifying bubble swarm patterns and heat transfer performance. The proposed general quantify model is defined as *η* = *a* ⋅ *γ* + *b* with 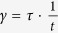. Here, *η* denotes heat transfer performance 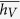, *a* denotes monomial coefficient, *b* denotes intercept term, *γ* denotes bubble swarm patterns, *τ* denotes the mean of index for measuring mixing and *t* denotes mixing time. In our previous work and this presented work, calculations confirm that Betti numbers^5^ and *S*^2^ both compliant with the same general pattern (Table 3). Apart from the two cases, we will also consider using the box-counting with erosions (considering *p*) method^2^ and the uniformity coefficient (considering *UC*) method^6^ to validate this general pattern (Table 3).

Three existed methods all can characterize the mixing performance. There is however no universally accepted technique for the particular technique issue mainly because each method has its own limitations. An original image-processing technique, the box-counting with erosions method, was developed for obtaining the quantification of mixing uniformity. It is however not available for quantifying the mixture nonhomogeneity. Betti numbers was proposed to quantify the mixture homogeneity and nonhomogeneity. Whereas, the distributions of mutliphase components or tracers in the image have relation with their geometrical position. The UC method was applied to address this issue in a direct contact heat exchanger. Mixing uniformity with the same Betti number can be identified with quite different uniformity coefficient in some very special cases.

**Figure 6:**
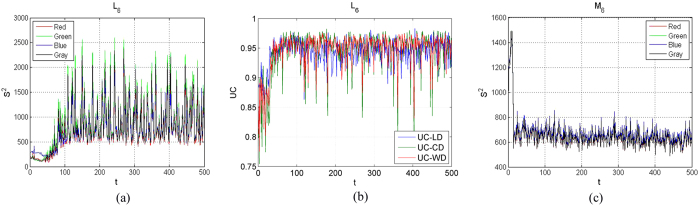
Evolution of *S*^2^ of the RGB components and gray scale values in our experimental cases: (**a**) applied the proposed new method to quantify the evolution of flow boiling bubbles; (**b**) applied UC-LD, UC-CD and UC-WD to quantify the evolution of flow boiling bubbles; (**c**) applied the proposed new method to a mixing system with top blown gas flow.

Also, they are limited by that the objective must be binary (black and white) image. The proposed statistical testing method in this work can be used to compute the evaluating value of mixture homogeneity in RGB components or gray images(Fig. 6a). It is noticed that *S*^2^ of the RGB components and gray scale value images exhibit a similar tendency. They all can be used to quantify the evolution of the flow boiling bubbles. Owing to the complexity of the multiphase structure, more useful information can be reduced as little as possible via this statistical approach. Simply, the estimate of error variance, *S*^2^, was proposed to characterize the mixing effect and the complex phase transition. Generally, the characterization of mixing uniformity by pixel values images can be elucidated. Hence, in this paper, the visualization of the flow pattern was investigated through the simple image processing technique and statistics theory. This work also presents an alternative route, namely builds a general model, to explore the relationship between the flow patterns and heat transfer from the viewpoint of experimental analysis.

Recently, uniformity coefficient based on centered discrepancy (UC-CD) and uniformity coefficient based on wrap-around discrepancy (UC-WD) were proposed for improving the UC method (UC-LD). They exhibit some advantages such as permutation invariance, rotation invariance (reflection invariance) and the ability to measure projection uniformity. The mixing time *t*_*CD*_ and *t*_*WD*_ were obtained at which UC-CD and UC-WD are equal to their averages, respectively(Table 1). Moreover, the average UC-CD 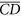 and *t*_*CD*_ were considered to validate this general model as well as the average UC-WD 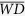 and *t*_*WD*_(Table 3).

### Examples

#### Tracers mixing

Testing for uniform lighting can also be very useful for studying the mixing efficiency and lighting uniformity in a mixing system with top blown gas flow. The numbers M_1_−M_9_ denote different experimental cases, *l* (cm) is the submerged length of the top-blowing pipe and *Q* (L/h) (liter per hour) is the flow rate of the gas (Table 4). Taking images regularly from the beginning of mixing to the end by above methodology, the evolution of *S*^2^ with time can be obtained. The inflection point *T*_*S*_(s) is approximately equal to the mixing time *T*_*B*_(s) obtained from the Betti numbers^4^ (Table 4).

**Figure 7:**
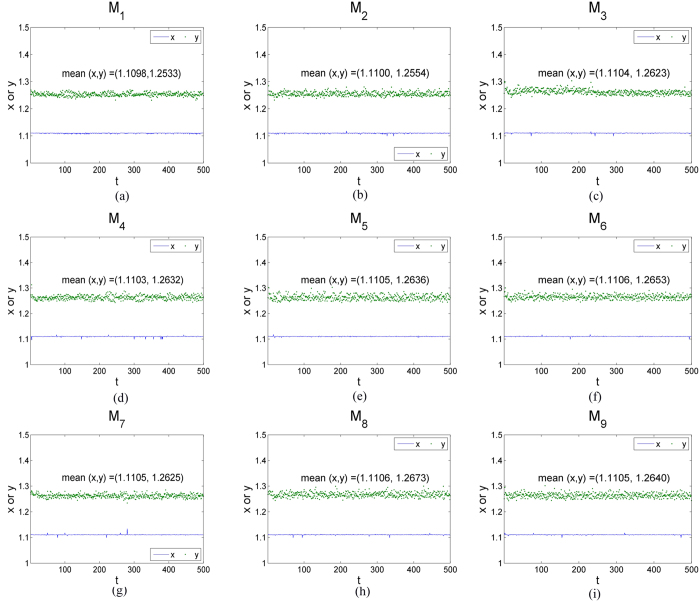
500 light source coordinates (*x*, *y*) with time obtained from images taken regularly from the beginning of mixture to the end in each experimental case M_1_–M_9_ and the values of coordinates smoothly oscillate about their averages (1.11, 1.26).

**Figure 8:**
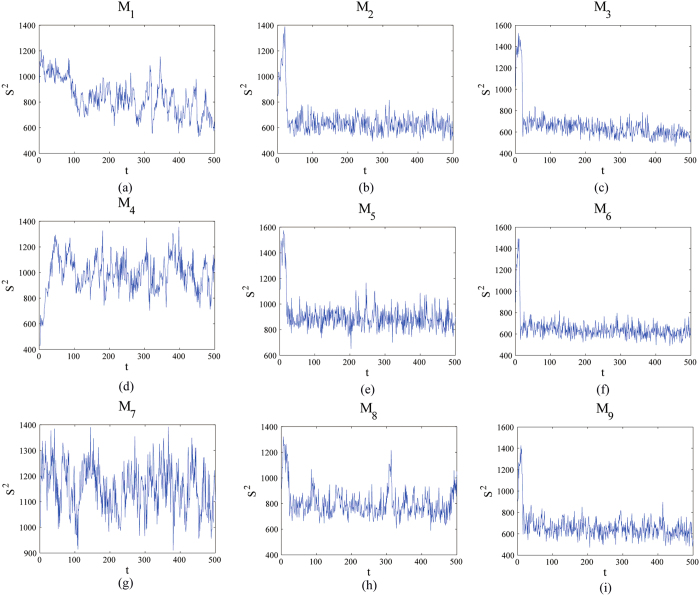
Evolution curves of the estimate of error variance, *S*^2^, in a mixing system with top blown gas flow and representative experimental design.

To show the potential of the presented statistical testing method, nine experimental levels M_1_-M_9_ were employed. All the representative experimental cases of the mixing system with top blown gas flow were chosen to be illustrated in Figs 7 and 8. It is evident that the final obtained location is not strictly fixed for every pattern because the variance of the random error is not identically equal (Fig. 7). Since the horizontal and vertical ordinate are both positive, the light comes from the top-left corner of the experimental equipment and the values of coordinates smoothly oscillate about their averages *x* = 1.11, *y* = 1.26 with the standard deviation of 0.028%, 0.46%, respectively. It is evident that M_6_ has the best mixing effect and M_1_ has the worst mixing effect (Fig. 8). It is clearly observed that *S*^2^ decreases at the beginning of M_6_ and then rapidly becomes stabilized after fluctuations. In addition, the *S*^2^ of M_1_ and M_7_ smoothly oscillated about their averages. Interestingly, the new index *S*^2^ decreases at the beginning of the mixing and then rapidly becomes stabilized after fluctuations. This means that that the two independent random variables, the abscissa *u* and the ordinate *v*, would change together decreasingly when multiphase components or tracers are visually distinguishable. Clearly, the final obtained location is not strictly fixed for every pattern because the variance of the random error is not identically equal.

#### Particles mixing

**Figure 9:**
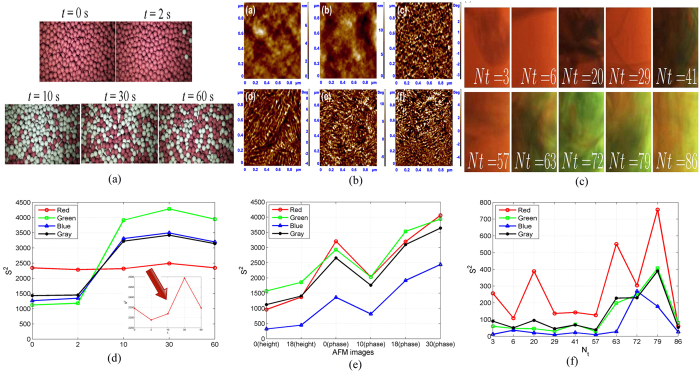
Evolution of *S*^2^ of the RGB components and gray scale values in applications: (**a**) particles mixing images reported by Liu *et al*.^17^; (**b**) AFM images reported by Gao *et al*.^18^; (**c**) images in the biorector reported by Rodriguez *et al*.^19^; (**d**) *S*^2^ of (**a**); (**e**) *S*^2^ of (**b**); (**f**) *S*^2^ of (**c**).

In a previous work, the RGB images of red (hot) and white (cold) particles mixing in a pan coaster during the first 60 s in test 1# (the white particles have the room temperature 20 °C while the red particles have a relatively high initial temperature 40 °C) was reported by Liu *et al*.^17^. The proposed methodology was applied to the RGB images of particle mixing on the bed surface (Fig. 9a). The RGB images were delimited by the Photoshop software. The values of *S*^2^ at t = 0 s, t = 2 s, t = 10 s, t = 30 s, t = 60 s were obtained by the MATLAB software, respectively. The time evolution of *S*^2^ of the RGB components and gray scale values was illustrated (Fig. 9d). The result indicates that *S*^2^ of the green, blue components and gray scale values increase during the time change, which is not the case with the red component. For the red component, at t = 2 s, only a few white (cold) particles reach the bed surface on which a majority of red (hot) particles are exposed; the value of *S*^2^ at t = 2 s described the bad particle mixing quantitatively. Moreover, the estimated average value of coordinates of light source is (−1969.13, 633.28). It was estimated that the light comes from the bottom-left corner since the horizontal ordinate is negative and the vertical one is positive at every time during the mixing process. This was verified visually.

#### Other applications

The methodology also can be used to study the polymer solar cells. The pending atomic force microscopy (AFM) images were delimited from previous investigation^18^ which was reported (Fig. 9b). The values of *S*^2^ the AFM height images of the poly(3-hexylthiophene) (P3HT): (C61-butyric acid methyl ester) PCBM blend films with and without solvent annealing, the AFM phase images of P3HT: PCBM blend films that were solvent annealed for 0, 10, 18, and 30 min are shown (Fig. 9e). It is noticed that *S*^2^ of the AFM height image is greater, which is also corresponded to the indicator of phase separation. Also, *S*^2^ of the phase images of P3HT:PCBM blend films exhibits an increasing tendency corresponding to the trend that the normalized interface length (*L*_*N*_) increases with increasing the solvent annealing time (*t*_*α*_) as a whole. Although light or electricity has no effect on AFM imaging basically, this could be useful to study the quantitative characterization of phase separation in the photoactive layer of polymer solar cells by the phase image of AFM. The last subgraph clearly shows the tendency of *S*^2^ of Fig. 3(b) in the literature^19^ and this is benefit for accurate determination of the mixing time in orbitally shaken bioreactor (Fig. 9f). According to Rodriguez *et al*.^19^, the white light was homogeneous. It is interesting to note that the variation of *S*^2^ exhibits fluctuations, which, as expected, can be used to study the mixing transient. In this case, owing to the complexity of the multiphase structure, it is difficult to convert into a black-white image to obtain a binary image by applying an appropriated grey level threshold for the colour video images. Experiments indicate that, *S*^2^ of the red component shows good correlation with the normalized red channels with time in the literature^19^. The absolute value of correlation coefficient is 0.6227. The feasibility of our method was verified from this application.

## Summary and Conclusions

In this study, the mixing uniformity influenced by a fixed light in a DCHE was investigated using an image analysis technique and a statistical method based on linear model. This method presents an alternative route to exploring the relationship between the flow patterns of bubble swarms influenced by fixed light and heat transfer from the viewpoint of experimental analysis. The summary and conclusions of this paper are as follows:

- The *F*-test was employed to conclude that the hypothesis that the gray distribution is uniformly distributed is overwhelmingly rejected.
- The direction of the light source was estimated correctly, validated by the previous design of the experiments. The accurate location of the light source, which has a significant effect on some specific experiments, such as sensitivity to light, was also determined and expressed by coordinate values in a coordinate system, which is the main merit of the proposed method.
- *S*^2^, the estimate of error variance, was newly proposed to quantify the flow patterns contaminated by light. The inflection point of its changing patterns approximately equals the mixing time.
- The parameter 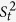, combined with *t* and 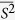, provides a good description to characterize the entire contaminated flow patterns. In particular, the experimental results showed a good fitting curve between 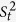 and 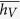. Hence, a general quantify model was first introduced to demonstrate their relationship.
- In addition, it can be of interest to the general public who wants to investigate the various relevant area in term of mixing process in which mutliphase components or tracers are difficultly or easily distinguishable by investigating RGB components or gray value images. In our experimental cases of flow boiling bubbles, the new index *S*^2^ increases at the beginning of the mixing and then rapidly becomes stabilized after fluctuations; In our experimental cases of a mixing system with top blown gas flow, the new index *S*^2^ decreases at the beginning of the mixing and then rapidly becomes stabilized after fluctuations. This means that that the two independent random variables, the abscissa *u* and the ordinate *v*, would change together increasingly when multiphase components or tracers are difficultly distinguishable whereas they would change together decreasingly when multiphase components or tracers are visually distinguishable. Hence, the topic of this manuscript used a simple visual method to perform unintrusive measurements or solve a complicated problem. Also, it can be applied in the relevant areas of powder technology, solar cells, biochemical engineering such as the particles mixing process in a pan coater, polymer solar cells, mixing time in the orbitally shaken bioreactors.

## Additional Information

**How to cite this article**: Xiao, Q. *et al*. Quantifying the evolution of flow boiling bubbles by statistical testing and image analysis: toward a general model. *Sci. Rep.* **6**, 31548; doi: 10.1038/srep31548 (2016).

## Figures and Tables

**Table 1:** The data of the parameters for the entire orthogonal array table.

Parameter	L_1_	L_2_	L_3_	L_4_	L_5_	L_6_	L_7_	L_8_	L_9_
*t*_*p*_	158	93	162	218	114	69	250	130	138
*t*_*B*_	156	93	168	225	122	84	262	117	128
*t*_*UC*_	151	97	172	224	120	84	259	118	127
*t*_*s*_	128	93	165	225	122	81	200	117	126
*t*_*CD*_	151	95	170	225	120	82	195	118	127
*t*_*WD*_	151	96	170	224	120	83	198	118	127
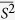	550	772	738	340	576	850	388	878	807
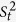	4.30	8.30	4.47	1.51	4.72	10.49	1.94	7.50	6.41
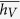	0.96	1.21	0.86	0.83	1.20	1.44	0.75	1.11	1.19

**Table 2:** Comparison of the *F*-test parameters for different image resolutions.

Image size	*α*_0_	*α*_1_	*α*_2_	*P*-value	*S*^2^	location
720 × 1280	132.03	−0.0527	−0.0001	0.0000	536.58	(1.23, 1.49)
360 × 640	131.77	−0.1054	−0.0003	0.0000	538.39	(1.14, 1.43)
180 × 320	131.91	−0.2110	−0.0015	0.0000	539.11	(1.06, 1.18)
90 × 160	132.01	−0.4223	−0.0035	0.0000	542.48	(0.80, 0.73)
45 × 80	132.52	−0.8451	−0.0148	1.0131e–156	545.94	(0.12, −0.03)

**Table 3:** The parameter and data of the *a*, *b* and correlation coefficient (cc).

Average index	reference	new index	*a*	*b*	cc
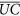	Fei *et al*.^6^	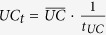	84.36	0.46	0.96
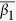	Huang *et al*.^5^	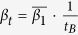	0.42	0.45	0.95
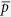	Coent *et al*.^2^	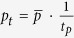	1801.06	0.61	0.90
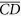	Xu *et al*.^2^	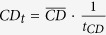	85.54	0.43	0.96
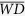	Xu *et al*.^2^	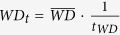	86.77	0.55	0.95
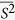	This paper	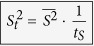	0.07	0.68	0.90

**Table 4:** Experimental parameters of top blown gas flow mixing system.

Parameters	M_1_	M_2_	M_3_	M_4_	M_5_	M_6_	M_7_	M_8_	M_9_
*l*(cm)	5	5	5	6	6	6	7	7	7
*Q*(L/h)	500	1000	2000	500	1000	2000	500	1000	2000
*T*_*B*_(s)	85.99	16.83	13.67	36.23	12.33	9.00	30.06	14.87	9.80
*T*_*S*_(s)	81.35	12.52	11.11 39.22	17.32	9.48	36.62	14.19	10.04	
